# Vaccine Development against Zoonotic Hepatitis E Virus: Open Questions and Remaining Challenges

**DOI:** 10.3389/fmicb.2018.00266

**Published:** 2018-02-19

**Authors:** Yuchen Nan, Chunyan Wu, Qin Zhao, Yani Sun, Yan-Jin Zhang, En-Min Zhou

**Affiliations:** ^1^Department of Preventive Veterinary Medicine, College of Veterinary Medicine, Northwest A&F University, Yangling, China; ^2^Scientific Observing and Experimental Station of Veterinary Pharmacology and Diagnostic Technology, Ministry of Agriculture, Yangling, China; ^3^Molecular Virology Laboratory, VA-MD College of Veterinary Medicine and Maryland Pathogen Research Institute, University of Maryland, College Park, MD, United States

**Keywords:** hepatitis E virus, zoonosis, HEV vaccine, subunit vaccine, quasi-enveloped virion, HEV-ORF3 product

## Abstract

Hepatitis E virus (HEV) is a fecal-orally transmitted foodborne viral pathogen that causes acute hepatitis in humans and is responsible for hepatitis E outbreaks worldwide. Since the discovery of HEV as a zoonotic agent, this virus has been isolated from a variety of hosts with an ever-expanding host range. Recently, a subunit HEV vaccine developed for the prevention of human disease was approved in China, but is not yet available to the rest of the world. Meanwhile, notable progress and knowledge has been made and revealed in recent years to better understand HEV biology and infection, including discoveries of quasi-enveloped HEV virions and of a new function of the HEV-ORF3 product. However, the impact of these new findings on the development of a protective vaccine against zoonotic HEV infection requires further discussion. In this review, hallmark characteristics of HEV zoonosis, the history of HEV vaccine development, and recent discoveries in HEV virology are described. Moreover, special attention is focused on quasi-enveloped HEV virions and the potential role of the HEV-ORF3 product as antibody-neutralization target on the surface of quasi-enveloped HEV virions to provide new insights for the future development of improved vaccines against zoonotic HEV infection.

## Introduction

Hepatitis E virus (HEV) is a single-stranded, positive-sense RNA virus which belongs to the genus *Orthohepevirus*, family *Hepeviridae* (Smith et al., 2014). HEV virions collected from fecal samples are non-enveloped spherical particles of approximately 27–34 nm in diameter (Meng, 2016). In humans, HEV infection generally causes self-limiting hepatitis with mortality generally ranging from 0.5 to 3%; however, up to 30% of infected pregnant women die if infection occurs in the third trimester of gestation (Jameel, 1999). For a long time, HEV was believed to be a public health concern only for humans in developing countries, due to poor sanitation and the predominant viral transmission mechanism via the fecal-oral route. However, the discovery of HEV infections of swine and other species suggests that the virus has a broad host range and is actually zoonotic (Christensen et al., 2008; Dalton et al., 2008; Meng, 2013; Pavio et al., 2015). Hepatitis E cases have been frequently reported in industrialized countries (Erker et al., 1999; Schlauder et al., 1999; Worm et al., 2000; Kabrane-Lazizi et al., 2001; Mizuo et al., 2002; Sadler et al., 2006). Consequently, as more HEV isolates with expanded host ranges have been confirmed (Smith et al., 2014), cross-species HEV infections have been more frequently recognized and are now considered to be the most important sources of virus for human infection in developed countries (Pavio et al., 2015; EFSA Panel on Biological Hazards (BIOHAZ) et al., 2017).

In recent years, both chronic HEV infection in immunocompromised patients and extrahepatic illnesses caused by HEVs have been documented (Kamar et al., 2011, 2012b; Hoofnagle et al., 2012; Grewal et al., 2014; van Eijk et al., 2014; Dalton et al., 2016; Geng et al., 2016). These findings suggest an underestimation of the importance of HEV as a public health concern, especially with regard to zoonotic HEV. Currently our understanding of HEV host range is limited in several key areas including non-foodborne transmission routes of zoonotic HEV from animals to humans, host tropisms of HEV, and viral determinants of HEV cross-species infection. Because effective vaccines are not yet available to prevent HEV infection, various treatments have been used to treat disease. For example, off-label use of ribavirin monotherapy for HEV has demonstrated certain therapeutic effects in both acute and chronic hepatitis E patients (Kamar et al., 2010; Mallet et al., 2010; Gerolami et al., 2011). However, viral resistance to treatment is a threat, as demonstrated by detection of ribavirin-resistant HEV mutants in patients (Lhomme et al., 2015; Todt et al., 2016). Indeed, ribavirin monotherapy was unable to cure chronic HEV infection in a persistently immunosuppressed patient (Miyoshi et al., 2016). Subsequently, application of pegylated interferon in combination with ribavirin was tested as a treatment for chronic HEV infection. However, the combined treatments only exhibited a moderately synergistic effect (Wedemeyer et al., 2012; Debing et al., 2014). More recently, other treatment formulations have been evaluated as anti-HEV therapeutics with promising results. One such treatment, peptide-conjugated morpholino oligomers (PPMOs), was developed as novel anti-HEV compounds in our laboratory (Nan et al., 2015). However, PPMOs are still far from ready for use in clinical applications. Therefore, vaccines still remain the best choice as an approach for achieving HEV prevention.

The goal to develop an anti-HEV vaccine can be traced back to soon after the initial discovery of HEV. However, HEV has been less investigated than other hepatic viruses. Consequently, much information has only recently become available to design HEV vaccine, including the discovery of zoonotic infection as well as detection of highly diverse HEV genotypes and newly identified quasi-enveloped HEV particles. Currently, only one HEV vaccine (Hecolin^®^) has been approved in China, while all other HEV vaccine programs have been discontinued. However, the most recent HEV discoveries have raised concerns that conventional subunit vaccine designs based on a single protein from a single viral strain might not confer protection against infection by various zoonotic HEV strains. In this review, the historical HEV vaccine pipeline and potential antigenic variation among zoonotic HEV isolates are discussed. In addition, the impact of newly identified quasi-enveloped HEV virions, as well as the potential role of HEV-ORF3 protein in HEV neutralization, is reviewed and perspectives and new insights are discussed.

## Genetic Structure of Hepatitis E Virus

The HEV genome is a 7.2-kb mRNA-like molecule which is capped and polyadenylated (Ahmad et al., 2011). Currently, three well-defied open reading frames (ORFs) have been identified for all HEV genotype with sequences encoding non-structural proteins designated by lower ORF numbers and higher-numbered ORFs coding for structural proteins (Tam et al., 1991; Tsarev et al., 1992). For genotype 1 HEV, additional ORF4 (within ORF1) is identified recently and its expression is driven by a putative internal ribosome entry site (IRES)-like sequence upstream of ORF4 that is only present within the genotype 1 HEV genome (Nair et al., 2016). The HEV genome contains two *cis*-reactive elements (CRE) that are essential for viral replication (Cao et al., 2010; Parvez, 2015). It is demonstrated that CRE located in the intergenic region of the HEV genome form a stem-loop structure that acts as a promoter-like element for RNA synthesis (Cao et al., 2010).

Since zoonotic HEV isolates are highly diverse, classification of HEV strains is currently in transition (Smith et al., 2013, 2014). In 2014, a newly proposed system of HEV classification within the family *Hepeviridae* was published as an attempt to formulate a unified classification scheme for this family (Smith et al., 2014). In this proposal, there are two genera, *Orthohepevirus* (including all mammalian and avian HEV isolates) and *Piscihepevirus* (only trout HEV isolates) within the family *Hepeviridae*. It is notable that all four previously known HEV genotypes (1–4) infecting humans are now known to belong to the species *Orthohepevirus A* (Smith et al., 2014), while the other three distinct species of *Orthohepevirus* (B, C, and D) include isolates from various non-human hosts (Smith et al., 2014). HEV genotypes 1 and 2 within *Orthohepevirus A* are restricted to humans with no known animal reservoirs, whereas genotypes 3 and 4 are zoonotic (Meng, 2010b; Ahmad et al., 2011). Moreover, within the well-defied HEV genotypes especially genotypes 1–4, there were several subtypes as well as clades existed (Smith et al., 2016).

Accordingly, two HEV-like viruses isolated from avian species and cutthroat trout that were previously referred to as avian HEV and cutthroat trout virus, respectively, have been classified in the new scheme as well. Avian HEV shares less than 50% nucleotide identity to mammalian HEV and is classified as species *Orthohepevirus B* (Haqshenas et al., 2001, 2002; Huang et al., 2004; Zhao et al., 2010; Smith et al., 2014). The cutthroat trout virus, which shares even lower sequence identity with mammalian than avian HEV, is classified as the only member of *Piscihepevirus* in this new proposed classification scheme (Batts et al., 2011; Smith et al., 2014). The classification of mammalian HEV genotypes, subtypes within each genotype, natural hosts, and their zoonotic features are summarized in **Table 1**.

**Table 1:** Classification of HEV genotypes and subtypes in relation to the virus hosts and disease development.

Genus	Species	Genotype	Proposed subtypes and reference sequence (Accession No.)	Natural hosts	Infectious to humans (Reference)	Reference of human infection	Chronic infection in immunocompromised person
*Orthohepevirus*	*Orthohepevirus A*	1	1a(M73218)	Human	Yes	Tsarev et al., 1992	No
			1b(D11092)				
			1c(X98292)				
			1d(AY230202)				
			1e(AY204877)				
			1f(JF443721)				
		2	2a(M74506)	Human	Yes	Reyes et al., 1991; Chatterjee et al., 1997	No
			2b(only partial sequence,AY903950)^1^				
		3	3a(AF082843)	Human, pig, rabbit, deer, mongoose, wild boar	Yes	Kwo et al., 1997; Meng et al., 1997; Schlauder et al., 1998	Yes
			3b(AP003430)				
			3c(FJ705359)				
			3d(only partial sequence, AF296165-7)				
			3e(AB248521)				
			3f(AB369687)				
			3g(AF455784)				
			3h(JQ013794)				
			3i(FJ998008)				
			3j(AY115488)				
		4	4a(AB197673)	Human, pig, yak, wild boar	Yes	Wang et al., 1999, 2000; Nishizawa et al., 2003	Yes
			4b(DQ279091)				
			4c(AB074915)				
			4d(AJ272108)				
			4e(AY723745)				
			4f(AB220974)				
			4g(AB108537)				
			4h(GU119961)				
			4i(DQ450072)				
		5	5a(AB573435)	Wild boar	Not Reported	Not Reported	N/A
		6	6a(AB602441)	Wild boar	Not Reported	Not Reported	N/A
		7	7a(KJ496143)	Camel	Yes	Lee et al., 2016	Yes
		8	N/A^2^	Camel	Not Reported	Not Reported	N/A
	*Orthohepevirus C*	C1	N/A	Rat	Unlikely based on non-human primate data	Not Reported	N/A
		C2	N/A	Ferret	Unlikely based on non-human primate data	Not Reported	N/A
	*Orthohepevirus D*	N/A	N/A	Bat	Not Reported	Not Reported	N/A

*^*1*^There is no complete genome sequence available for corresponding GenBank accession number. ^*2*^Data for corresponding information is not available or not yet tested according to the available literatures*.

Meanwhile, with the continual discovery of new HEV or HEV-related isolates from rat, ferret, bat, moose, farmed mink, camel, and wild boar (Lorenzo et al., 2007; Zhao et al., 2009; Johne et al., 2010; Geng et al., 2011; Takahashi et al., 2011; Drexler et al., 2012; Raj et al., 2012; Krog et al., 2013; Lin et al., 2014; Woo et al., 2014), new genotypes have been assigned to these new isolates. Several wild boar HEV isolates from Japan with unique sequences have been assigned to genotypes 5 and 6, while HEVs from camel have been classified as genotypes 7 and 8 of *Orthohepevirus A* (Smith et al., 2014; Woo et al., 2016).

HEV-ORF1 is translated directly from its mRNA-like genome and encodes all non-structural proteins (mainly replicase) required for HEV replication. The functional domains within the HEV-ORF1 polyprotein include an RNA capping enzyme domain, a papain-like cysteine protease, a hypervariable region, a macro domain, an RNA helicase domain, and an RNA-dependent RNA polymerase domain (RdRp) (Ahola and Karlin, 2015; Nan and Zhang, 2016). The hypervariable region of HEV-ORF1 is an intrinsically disordered region (IDR) that is involved in gene segment insertion or deletion (Purdy, 2012; Purdy et al., 2012). Notably, this region might be involved in creation of recombinant viruses containing genetic elements from various HEV strains or even other hosts (Shukla et al., 2011). Moreover, the observed swapping of ORF1 sequences between genotypes 1 and 4 HEV infectious clones indicates that the ORF1 product may participate in cross-species infection and influence HEV host tropism (Chatterjee et al., 2016).

**FIGURE 1:**
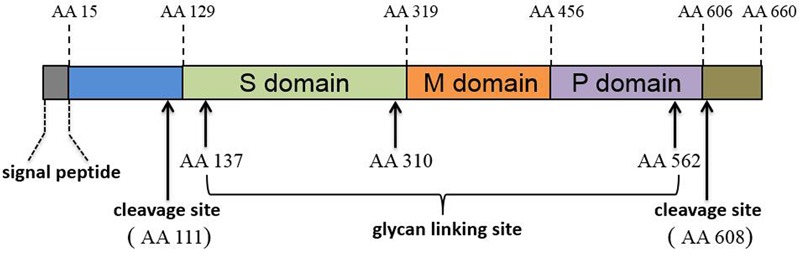
Schematic illustration of HEV-ORF2 protein domains.

The ORF2 and ORF3 products were determined via protein sequence prediction from sub-genomic RNA sequences and these proteins partially overlap with one another (Graff et al., 2006). ORF2 encodes the capsid protein (the major component of the HEV virions). The mature HEV capsid protein requires proteolytic processing and the final product, lacking the first 111 amino acid (aa) and the last 52 aa of full-length ORF2 product, forms virus-like particles (VLP) when expressed in a baculovirus-based system (Li et al., 1997, 2005c). Crystal structure determinations suggest that HEV capsid protein has three domains designated shell (S, aa 129–319), middle (M, aa 320–455), and protruding (P, aa 456–606) (Yamashita et al., 2009) (**Figure 1**). Genetic analysis of ORF2 has demonstrated over 85% similarity among genotypes 1–4 HEV strains that infect humans (Mori and Matsuura, 2011). Moreover, the identification of both conformational and linear neutralizing epitopes in HEV capsid protein suggests that this protein is the target for neutralizing antibodies (Gu et al., 2015; Tang et al., 2015). Consequently, most HEV vaccine candidates are based on recombinantly expressed full length or truncated HEV-capsid proteins. Meanwhile, HEV-ORF3 protein encodes a small but multifunctional protein that is indispensable for HEV infection *in vivo* (Graff et al., 2005; Huang et al., 2007; Mori and Matsuura, 2011). The function of HEV-ORF3 is less well understood, but the majority of studies indicate that it is involved in virus release from cells and formation of newly identified “quasi-enveloped” HEV virions in infected cells (Hurley, 2010; Nagashima et al., 2011a,b, 2014; Feng et al., 2014; Yin et al., 2016). The ORF3 product has recently been suggested to be a viroporin that forms a functional ion channel involved in the release of infectious HEV virions (Ding et al., 2017).

Recently, a novel ORF4 was predicted from HEV genotype 1 sequences (Nair et al., 2016). However, it appears that this product is produced only in genotype 1 HEV, as its expression is driven by a putative IRES-like sequence upstream of ORF4 that is only present within the genotype 1 HEV genome (Nair et al., 2016).

## Hepatitis E Virus as a Zoonotic Pathogen

Soon after the identification of HEV as the causative pathogen for hepatitis E, HEV was regarded as a viral pathogen restricted only to humans. This supposition was supported by the fact that no other hepatic viruses (HAV, HBV, and HCV) were known to be zoonotic except for experimental infections of non-human primates by above hepatic viruses (Purcell and Emerson, 2001; Chayama et al., 2011). However, these assumptions conflicted with the finding that HEV antibodies were found in a surprisingly high proportion of the population (up to 28% in some areas) in the United States and other developed countries where hepatitis E was not thought to be endemic (Mast et al., 1997; Thomas et al., 1997). This finding suggests that unknown sources of HEV infection exist or that unrecognized non-pathogenic HEV strains endemic to developed countries circulate undetected. Previously it had been reported that domestic swine were susceptible to experimental infection with a human HEV strain of unknown genotype (Balayan et al., 1990). Meanwhile, several other reports indicated that HEV antibodies naturally existed in primates and swine and demonstrated that a proportion of swine shed HEV RNA in stools (Clayson et al., 1995; Meng et al., 1997). Moreover, detection of anti-HEV antibodies was documented in a number of other animal species, such as sheep and rats (Karetnyi Iu et al., 1993; Usmanov et al., 1994; Maneerat et al., 1996; Meng et al., 1998). After the characterization of HEV, experimental infections of HEV in rats and lambs were attempted to develop animal HEV infection models using human HEV isolates of unknown genotypes, but demonstrated mixed results (Usmanov et al., 1994; Maneerat et al., 1996). Therefore, results of these earlier reports suggested that HEV might be a zoonotic pathogen, even though cross-species infectivity of HEV was not yet confirmed during that time.

The hallmark of zoonotic HEV research was the identification of swine HEV closely related to the human hepatitis E virus circulating in swine herds in the mid-western United States (Meng et al., 1997). Simultaneously, HEV strains US-1 and US-2 recovered from two acute clinical cases of hepatitis E were quite distinct from previously recognized human HEV strains, but were closely related to swine HEV isolated in 1997. US-1 and US-2 share more than 97% aa identity with swine HEV, providing genetic evidence of HEV zoonosis (Kwo et al., 1997; Meng et al., 1998; Schlauder et al., 1998). Subsequently, experimental inoculation of rhesus monkeys, chimpanzees, and specific-pathogen-free (SPF) pigs with these two human HEV isolates demonstrated that swine HEV can cause cross-species infection. Therefore, it should be emphasized here that humans appear to be at risk for infection with swine HEV (Meng et al., 1998).

Since the mid-1990s, numerous HEV isolates have been identified from various animal species. The presence of HEV in domestic pig and wild boar populations has been confirmed in both developing and developed countries (Meng et al., 1997; Meng, 2010a; Thiry et al., 2014). Meanwhile, rabbit HEV was detected in 2009 in China, the United States, France, and United Kingdom (Zhao et al., 2009; Cossaboom et al., 2011; Izopet et al., 2012; Ijaz et al., 2013; Grierson et al., 2015; Liu et al., 2017). It appears that foodborne pathways of HEV from animal reservoirs to human act as the major transmission routing of HEV in developed countries since acute hepatitis E infections have been linked to consumption of contaminated pork or pig liver (Kamar et al., 2013; Pavio et al., 2014; Cossaboom et al., 2016; EFSA Panel on Biological Hazards (BIOHAZ) et al., 2017). Based on surveillance studies, approximately 2% of pig livers sold in Japan and 11% of pig livers sold in the United States are positive for HEV-RNA (Yazaki et al., 2003; Feagins et al., 2007). In another investigation from United Kingdom, present of HEV antibodies and RNA in serum and cecal contents of pigs at the time of slaughter were up to 92.8 and 15%, respectively (Grierson et al., 2015). Based on the report of European Food Safety Authority, HEV prevalence in pig production regions in Europe, as well as within herds of domestic swine, is often very high (98%, 204/208 positive herds in Spain; 55% of 97 herds positive in Netherlands EFSA Panel on Biological Hazards (BIOHAZ) et al., 2017).

Studies have also suggested that humans appear to be at risk of infection from rabbit HEV as well (Zhao et al., 2009; Lhomme et al., 2013; Liu et al., 2013; Xia et al., 2015). Other studies have suggested that rabbit HEV (genotype 3 or HEV-3) is capable to infect non-human primates and pigs to induce hepatitis (Liu et al., 2013; Han et al., 2014), which has the potential to be zoonotic HEV virus as well (Park et al., 2016). Notably, it appears that no major adaptation to new host of HEV-3 is required when transmitting between different hosts, since virus recovered from non-human primates infected with rabbit HEV exhibited 99.8% nucleotide sequence identity with original rabbit HEV (Pavio et al., 2015). Remarkably, research from our laboratory has demonstrated that rabbits from a SPF rabbit vendor exhibited a high positive rate of HEV antibodies (Liu et al., 2017).

Subsequently, the identification of swine and rabbit HEV has stimulated further research to investigate the serological prevalence of HEV in other animal species (Dong et al., 2011). As a result, ruminants such as cattle, sheep, and goats, as well as carnivores such as cats and dogs, have all exhibited the presence of HEV antibodies. Therefore, HEV transmission may be associated with human-pet contact (Liang et al., 2014). In addition to these hosts, HEV or the HEV genome have been isolated from such diverse species as chicken, mongoose, bat, ferret, rat, camel, trout, and wild boar (Smith et al., 2015). Therefore, it is likely that the host range of HEV will continually expand as novel strains continue to be identified.

## Development of an HEV Vaccine: a Two-Decade-Long Effort that is Still Ongoing

Cell culture systems have greatly facilitated development of numerous vaccines. However, until now available cell culture systems for HEV have not been robust, creating a major obstacle that has impeded HEV research and vaccine development. One culture system using S10-3 cells, a subclone of Huh7 hepatoma cells, supported improved replication efficiency of genotype 1 HEV strain Sar-55 above that of its Huh7 parental cell line. However, this system relies on the transfection of full-length HEV RNA (Graff et al., 2006; Shukla et al., 2011). Alternatively, another cell culture system using the A549 cell line that does not require RNA transfection supported only limited replication of genotype 1 HEV from a patient serum sample (Takahashi et al., 2010). Meanwhile, several other groups have demonstrated that genotype 3 or 4 HEV strains can be adapted to cell culture and gain the ability to infect cells. Although several HEV cell culture systems and cell-adapted HEV strains have been reported (Tanaka et al., 2007; Okamoto, 2011; Berto et al., 2013; Johne et al., 2016; Nan and Zhang, 2016), application of these HEV *in vitro* models for generation of inactivated vaccine or modified live virus (MLV) vaccines is not reported yet.

Fortunately, the capsid protein of HEV shares over 85% identity with all four major HEV genotypes infecting human or other mammalian hosts. Therefore, ORF2 is a good HEV subunit vaccine candidate (Mori and Matsuura, 2011) and prompted the development of recombinant HEV capsid proteins as subunit vaccines beginning soon after the discovery of HEV (Purdy et al., 1993). Unfortunately, most HEV vaccine programs based on recombinant HEV capsids were discontinued at the pre-clinical stages due to mixed results or incomplete protection.

In spite of early failures, researchers have employed numerous other strategies to create better ORF2-based vaccines. One such strategy employed bacterially expressed trpE-C2 fusion protein (containing 221–660 of ORF2 of the genotype 1 HEV Burmese strain) as the first recombinant protein HEV vaccine (Purdy et al., 1993). Preliminary data suggested that the vaccine completely protected cynomolgus macaques challenged with a homologous HEV Burmese strain. However, in vaccinated animals challenged with a Mexican strain of HEV genotype 2, virus shedding and HEV antigen were detected in stools and liver, respectively (Purdy et al., 1993). Since then, additional expressed recombinant ORF2 products or truncated ORF2 products have been tested for vaccine potential. In China, the first tested recombinant HEV-ORF2 protein vaccine employed a truncated ORF2 protein (aa 394-607 of ORF2 of a Chinese genotype 1 HEV isolate) expressed in *E. coli*. However, this recombinant vaccine only worked in rhesus macaques immunized with three 100 μg doses in Freund’s adjuvant and was poorly immunogenic in mice when administered with alum adjuvant (Li et al., 2005b). Next, the same group focused on improvement of ORF2 truncation and selected a 239 aa truncated ORF2 region (HEV239, aa 368-606 of the ORF2 product), which was more immunogenic than the aa 394-607 ORF2 truncated product (Li et al., 2005a,b). After HEV239 was tested in a clinical trial in humans, it was approved in China in 2012 as the only HEV vaccine available worldwide under the new trade name Hecolin^®^. These results thus suggest that the development of an effective ORF2-based HEV subunit vaccine may not be as straightforward as researchers had initially predicted (WHO, 2015).

Using a different strategy, VLP have also been tested for effectiveness as HEV vaccines. HEV encodes a single structural protein that is necessary for VLP production. Indeed, expression of ORF2 fragment (aa 112-608, genotype 1 HEV Burmese strain, 50 kDa in size) forms VLPs (Li et al., 1997, 2005c). Oral administration of five 10 mg doses of VLPs can confer protection from hepatitis for immunized macaques upon challenge with 10,000 50% monkey infectious dose (MID_50_) of homogenous genotype 1 strain, resulting in only one observed case of viral shedding (Li et al., 2004). Another VLP type HEV vaccine was developed based on the genotype 1 HEV Sar-55 strain, but the results were inconclusive. When expressed in insect cells, the full-length ORF2 of the Sar-55 strain produced four ORF2-related peptides with sizes of 72 (full length), 63, 56, and 53 kDa (Robinson et al., 1998). Unlike the genotype 1 Burmese strain, the 53 kDa product (aa 112-578 of ORF2, the smallest product) was able to form VLPs as well (Robinson et al., 1998). By contrast, the 56 kDa ORF2 product (aa 112-607 of Sar-55-ORF2) did not form VLPs nor did the comparable ORF2 region of the Burmese strain (Robinson et al., 1998). It is still unclear why the 53 kDa ORF2 fragment was generated and also why the 56 kDa Sar-55 ORF2 fragment could not form VLP. Nevertheless, VLPs formed from this fragment, due to the lack of a neutralizing epitope located in aa 578–607, could not induce full protective immunity but did reduce virus shedding in rhesus macaques after high-dose challenge (Zhang et al., 2001). Meanwhile, immunization with two doses of 56 kD Sar-55-ORF2 with alum adjuvant conferred protection against hepatitis in rhesus monkeys upon intravenous challenge with 10,000 plaque-forming units as the MID_50_ of either the homologous genotype 1, heterogeneous genotype 2, or genotype 3 HEV strain (Purcell et al., 2003). As a result, recombinant 56 kDa Sar-55 ORF2 was further evaluated in human clinical trials. However, it is somewhat surprising that results of studies using cleaved ORF2 products for a given HEV genotype were inconsistent among different research groups. As an example, 53 and 50 kDa ORF2 cleavage products were not observed during expression of the ORF2 fragment (aa 112–660, genotype 1 HEV Burmese strain) in insect cells. Instead, two ORF2-related polypeptides of genotype 1 HEV Burmese strain with sizes of 73 and 62 kDa were produced when ORF2 was expressed in a baculovirus system (McAtee et al., 1996). Therefore, it appears that the steps of proteolytic processing and assembly of ORF2 cleavage products to generate VLPs differ greatly among diverse systems. The reasons underlying these differences, as well as variations in immune responses evoked by various ORF2 cleavage products, remain to be investigated.

Besides prokaryotic and eukaryotic expression-based ORF2 subunit vaccines, DNA vaccines based on full or partial HEV-ORF2 sequences have also been evaluated in cynomolgus macaques. The first such vaccine, designated pcHEVORF2, contained the full ORF2 sequence of HEV genotype 1 Burmese strain. This vaccine was administrated intramuscularly to macaques and was shown to stimulate HEV-specific antibodies in immunized animals (Kamili et al., 2002). However, protection was only observed in half of the animals challenged with a heterologous genotype 2 Mexican HEV strain (Kamili et al., 2002). The second DNA vaccine was a liposome-encapsulated DNA (20 μg) mixed with peptide expressed in *E. coli* (20 μg, corresponding to a neutralizing epitope mapped to ORF2 aa 458–607) which is based on an Indian genotype 1 strain (Arankalle et al., 2009). After immunization, macaques were fully protected from challenge with 10,000 copies of HEV RNA from a homologous strain (Arankalle et al., 2009). Meanwhile, another novel vaccine candidate has been developed based on *E. coli* expression of the ORF3 of genotype 4 HEV as a fusion protein with interleukin-1β (Ma et al., 2009). This ORF3-based vaccine only induced partial protection, but was able to reduce the virus titer in blood, decrease fecal shedding of virus, and shorten the duration of viremia (Ma et al., 2009). The protective effects of this ORF3-based HEV vaccine are consistent with the recent characterization of “quasi-enveloped” HEV virions, which contain ORF3 product and are further discussed below (Yin et al., 2016; Nagashima et al., 2017).

Although a variety of methods have been used for HEV vaccine development in animals to date, in China, only two programs were eventually approved for clinical trials using human subjects, with only one program approved at this time (WHO, 2015). The first vaccine, the baculovirus-expressed 56 kDa truncated HEV-ORF2 protein (genotype 1 Sar-55), is well-tolerated and highly immunogenic in humans, achieving 95% protection efficacy in a phase II trial (Shrestha et al., 2007). However, this vaccine program has since ended. The second vaccine, HEV239 (under the trade name Hecolin^®^), which was finally approved and commercialized in China, is a truncated protein (aa 368–606 of ORF2) expressed in *E. coli* (Li et al., 2005b). After 4.5 years of follow-up, phase III trial results suggested that HEV239 was well tolerated and exhibited 100% protection from hepatitis E after administration of three immunization doses over 6 months (Zhu et al., 2010; Zhang J. et al., 2015). In summary, Hecolin^®^ is the only HEV vaccine available worldwide, but is only available commercially in China.

## Concerns About Antigenic Variation of Capsid Proteins Encoded by HEV-ORF2

Although Hecolin^®^ demonstrated promising protection from HEV infection in human trials, several concerns still exist. Hecolin^®^ was developed based on a genotype 1 Chinese HEV strain and should therefore confer the highest level of protection against genotype 1 HEV (Li et al., 2005b). As demonstrated in other pre-clinical evaluations of HEV vaccine candidates, genotype 1-based recombinant vaccines have exhibited highly effective protection against homologous challenge. These results may be partially due to the fact that genotype 1 HEV is the most conserved among all HEV genotypes (Nan and Zhang, 2016).

As mentioned above, genotype 1 HEV is restricted to humans and thus has no known animal reservoirs (Lu et al., 2006; Meng, 2010b; Ahmad et al., 2011). Therefore, improvement of sanitation conditions has been a highly effective strategy to block fecal-oral transmission of genotype 1 HEV. However, although genotype 1 HEV has been responsible for most previously documented endemic and epidemic cases of hepatitis E in Asia, most HEV cases in recent years have actually been caused by genotypes 3 and 4 HEVs (Kamar et al., 2012a). Therefore, the number of people best benefiting from Hecolin^®^ immunization (people with potential risk of genotype 1 HEV infection) is likely only a subset of potential cases at present.

Because most reported HEV cases in recent years have been caused by zoonotic HEV (genotypes 3 and 4), for maximum protection an effective HEV vaccine should protect recipients against zoonotic HEV rather than against human HEV only (genotypes 1 and 2) (Nan and Zhang, 2016). The high homology (85% identity) of ORF2 sequences among all four major HEV genotypes infecting humans increases the likelihood of achieving cross-protection through the administration of Hecolin^®^. However, it remains unknown whether or not Hecolin^®^ would provide full protection against all human or animal zoonotic HEV infections and therefore prevent foodborne zoonotic HEV transmission. Indeed, several studies have demonstrated that Hecolin^®^ vaccine could protect rabbits against genotype 4 HEV challenge (Liu et al., 2014; Zhang Y. et al., 2015).

As discussed in above section, there is strong evidence demonstrating that antigenicity of capsid proteins differs between genotypes 1 and 2 HEV (Purdy et al., 1993; McAtee et al., 1996; Yarbough, 1999; Kamili et al., 2002). For zoonotic HEV or animal vaccine candidates, few reports describe protection efficiency of subunit vaccines. Some reports demonstrate that convalescent sera from animals infected with any of genotypes 1–4 of HEV are capable of neutralizing genotype 1 HEV *in vitro* (Emerson et al., 2006a). Conversely, one study evaluated cross-protection against heterologous HEV after vaccination of pigs with truncated ORF2 capsid proteins (aa 112–660) derived from swine, rat, and chicken HEV. Subsequently, only partial protection was achieved against a genotype 3 mammalian HEV (Sanford et al., 2012). Meanwhile, in our previous report, rabbit antiserum against *Orthohepevirus B* (avian HEV) derived ORF2 peptide (aa 389–399) recognized only human HEV-ORF2 protein (Guo et al., 2006), further emphasizing that antigenic variation of various HEV genotypes may be much more diverse than originally assumed. Moreover, in a more detailed study to evaluate antibody cross-reactivity against capsid proteins of various HEV genotypes, the putative neutralizing region (aa 452–617) of ORF2 products of various HEV genotypes was employed in an indirect ELISA to measure patient sera reactivity toward ORF2 aa 452–617 from various HEV genotypes (Behloul et al., 2015). Based on the results, patient sera exhibited variable immune reactivity against the four antigens tested (Behloul et al., 2015). Ultimately, it appears that the aa 483–533 region demonstrated the highest antigenic potential; however, it contained an incomplete putative neutralizing domain, as deduced from results of previous studies of other vaccine candidates (Zhang et al., 2001; Behloul et al., 2015). Meanwhile, results from immunization of animals with Hecolin^®^ (p239) and another truncated p179 protein (based on ORF2 of genotype 4 HEV) showed that Hecolin^®^-induced IgGs produced in mice and humans reacted strongly with genotypes 1 and 2 recombinant ORF2 proteins. However, p179-induced IgGs from mice exhibited stronger reactivity to ORF2 proteins of genotypes 3 and 4 rather than for genotypes 1 and 2 ORF2 products (Wen et al., 2016). Similar differences have also been observed using convalescent sera of rhesus monkeys infected with genotype 1 or 4 HEV strains (Wen et al., 2016), which suggests the presence of distinct genotype-specific neutralizing epitopes among the HEV genotypes (Wen et al., 2016). Additionally, research from our laboratory also identified two epitopes and other antigenic domains shared by avian, swine, and human HEVs (Wang et al., 2015; Zhao et al., 2015). Thus, it appears these two epitopes are not protective epitopes and are unable to mediate viral neutralization. Therefore, such consensus epitopes among human and zoonotic HEV isolates ultimately might not be protective enough to confer complete cross-protection against heterologous HEV (Wang et al., 2015; Zhao et al., 2015).

Considering the heterogeneous nature of zoonotic HEV isolates, especially of genotypes 3 and 4 isolates, it is questionable that the 85% homology among capsid proteins of zoonotic HEVs is sufficiently high enough to mediate cross-protection if vaccine recipients only receive a single strain-based recombinant vaccine. This concern is bolstered by studies of another virus, a swine RNA virus known as porcine reproductive and respiratory syndrome virus (PRRSV). In this example, neutralizing antibodies to the vaccine strain of MLV were unable to both neutralize virus and did not achieve protection against heterogeneous strains that share homology of as high as 86% with the vaccine strain (Nan et al., 2017). Taken together, these results show that is premature to conclude that genotype 1 HEV-based Hecolin^®^ is efficacious enough to meet all practical requirements for effective zoonotic HEV prevention and control.

## Improved Zoonotic HEV Vaccines: Implications From Quasi-Enveloped HEV Virions

### Discovery of “Quasi-Enveloped” HEV Virions

As mentioned above, HEV has traditionally been classified solely as a non-enveloped virus. The presence or absence of an envelope has traditionally been used to classify viruses. This distinction was based on the sensitivity of enveloped virus infectivity to bile salts exposure, which removes the envelope lipid layer on the outside viral surface and abrogates infectivity (Feng et al., 2014). Generally, a viral envelope is formed by a budding process from membranes of infected cells, with at least one virus-encoded glycoprotein (peplomer) embedded in this layer. Such viral glycoproteins or other viral proteins embedded in the envelope mediate diverse interactions with cellular receptors expressed on susceptible cells and facilitate the fusion of cellular and viral membranes following initial virus-receptor interactions. For the host, viral glycoproteins on the envelope surface may also serve as antibody-neutralization targets (Feng et al., 2014). However, the viral envelope protects internal antigens (i.e., nucleocapsid) from being recognized by neutralizing antibodies (Putz et al., 2006). Therefore, a given quasi-enveloped virus would appear antigenically distinct from its non-enveloped counterpart by the host immune system.

Virus envelopes confer both advantages and disadvantages. On the one hand, an envelope can facilitate virus release and trafficking out of cells, while release of non-enveloped viruses relies on lysis of infected cells (Putz et al., 2006; Yin et al., 2016). On the other hand, the envelopes of such viruses are susceptible to environmental factors such as drying or exposure to solvents and detergents. Therefore, transmission of enveloped viruses requires relatively close contact with host cells for infection, achieved by inhalation of moist aerosols or exchange of secretions (Putz et al., 2006). By contrast, non-enveloped viruses spread readily through the environment and maintain infectivity under harsher conditions to cause food or waterborne diseases, including HAV and HEV infections (Lemon, 1985; Purcell and Emerson, 2008).

The HAV was the first non-enveloped virus later confirmed to possess an enveloped form derived from the host cell membrane (Feng et al., 2013). The biogenesis of enveloped HAV particles depends on a system comprised of endosomal sorting complexes required for transport (ESCRT) (Feng et al., 2013; Votteler and Sundquist, 2013). Membrane-wrapped HAV particles mainly circulate in blood during acute infection, whereby the envelope protects the virus from recognition of neutralizing antibodies without impairing virion infectivity (Feng et al., 2013). Similarly, membrane-wrapped HEV particles were confirmed as well. An earlier study of HEV particles isolated from hepatitis E patient sera had demonstrated that serum HEV virions could infect cells despite the presence of anti-HEV antibodies (Takahashi et al., 2010). In addition, an immune-capture assay of HEV virions treated with or without detergent indicated that HEV particles from both serum samples or cell culture supernatants were associated with lipids and HEV-ORF3 protein (Takahashi et al., 2010). Moreover, it was also demonstrated that both the tumor susceptibility gene 101 (Tsg101) and the vacuolar protein sorting pathway were required for the release of hepatitis E virions (Nagashima et al., 2011b). These observations are consistent with the putative function of HEV-ORF3, since the interaction between HEV-ORF3 protein and Tsg101, an ESCRT complex component often involved in enveloped virus budding, induces biogenesis of membrane-associated “quasi-enveloped” HEV particles (Hurley, 2010; Feng et al., 2014; Nagashima et al., 2014; Yin et al., 2016). As additional evidence, the most recent study demonstrated that quasi-enveloped HEV particles are released from cells via an exosomal pathway (Nagashima et al., 2017).

### Quasi-Enveloped HEV Particles: A New Strategy for Immune Escape by Non-enveloped Virus

**FIGURE 2:**
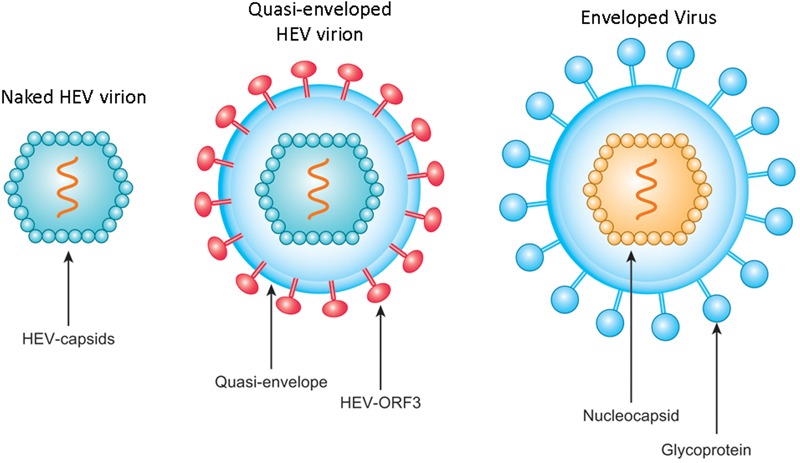
Schematic illustration of non-enveloped and quasi-enveloped HEV particles as well as enveloped virus. The putative model of quasi-enveloped HEV virion includes ORF3 product in its envelope as the existence of pORF3 has been confirmed by capturing quasi-enveloped HEV virion with anti-pORF3 antibodies and further supported by prediction of a putative transmembrane region in the N-terminal of pORF3.

As a fecal-orally transmitted agent, HEV is shed during passage through the gut and is subsequently found in high titers in feces of infected individuals as stable, non-enveloped virions with capsid-encapsulated genomes (Feng et al., 2014; Nan and Zhang, 2016). By contrast, the low yield of HEV from cell culture systems results in an inadequate supply of virus particles for experimental study (Okamoto, 2013). For this reason, most researchers studying HEV did not detect quasi-enveloped HEV particles until recently (Yin et al., 2016; Nagashima et al., 2017), with such particles reported only for HAV and HEV to date (Feng et al., 2013; Yin et al., 2016; Nagashima et al., 2017). Subsequently, incubation of HEV-ORF2 antibodies with quasi-enveloped HEV virus (**Figure 2**) suggested that quasi-enveloped virions which were unable to bind antibodies against the capsid protein (Takahashi et al., 2010), which implies that the majority of HEV virions released into sera as quasi-enveloped form and is inaccessible to HEV neutralizing antibodies that employ HEV-ORF2 as a primary target.

Additional research is needed to establish whether quasi-enveloped HEV virions from all HEV genotypes can be generated in infected hosts, as observed using current cell culture systems for propagation of genotype 3 or 4 HEVs (Yin et al., 2016; Nagashima et al., 2017). Because the PSAP motif (aa 96–99) of HEV-ORF3 is required for virion release and is therefore associated with biogenesis of quasi-enveloped HEV virions, it is important to note here that this epitope is conserved among all four major genotypes of HEV infecting humans and avian HEV, as well (Emerson et al., 2010; Nagashima et al., 2011a; Kenney et al., 2012). Indeed, the conservation of this epitope implies that quasi-enveloped HEV virions are common among all HEV genotypes, including zoonotic HEV isolates. Challenge experiments conducted to evaluate vaccine candidates in animals (including our own avian HEV vaccine studies), and most challenge viruses were collected from stool suspensions as a convenient source of high-titer HEV (Purdy et al., 1993; Zhang et al., 2001; Purcell et al., 2003; Guo et al., 2007; Zhou et al., 2008). In experiments evaluating Hecolin^®^ protection efficiency against zoonotic HEV in rabbits, challenge virus also originated from fecal samples (Liu et al., 2014; Zhang Y. et al., 2015). Since HEV virions collected from stool samples are naked and lack envelopes, such challenge viruses are highly susceptible to neutralization by conventional vaccine-elicited antibodies to ORF2 capsid antigens. Therefore, the protective efficacy of these vaccine candidates during natural infection might actually be lower than predicted using naked challenge virus from stools. Nevertheless, even under optimal circumstances, the cross-protective function of such vaccine candidates against strains of heterologous genotypes may still lack the necessary efficacy to meet the practical needs discussed above.

Importantly, most of the reported sporadic hepatitis E cases include foodborne infections caused by uncooked/undercooked products from infected animals, person-to-person blood transfusion-mediated infections, and solid organ transplant-mediated HEV transmission events (Pas et al., 2012; Wedemeyer et al., 2012; Pauli et al., 2015; Sue et al., 2016). These HEV transmission routes minimize *ex vivo* environmental influences, speculation that HEV particles causing infection in these cases are quasi-enveloped viral particles rather than naked particles. Exposure to *ex vivo* environmental factors such as detergents and proteases can remove the lipid component from quasi-enveloped particles to make them accessible to antibodies (Qi et al., 2015). Therefore, it remains to be determined whether or not vaccine-induced antibodies possess adequate capability to neutralize such quasi-enveloped HEV particles *in vivo* to confer disease protection. In the case of HAV, it appears that anti-capsid antibodies only restrict viral replication after infection with quasi-enveloped HAV *in vitro* (Feng et al., 2013). Therefore, such results suggest that capsid-based HEV vaccines might not be able to prevent a first-round infection by quasi-enveloped HEV.

### The HEV-ORF3 Product As Potential Target for Viral Neutralization of Quasi-Enveloped HEV Particles

With regard to quasi-enveloped HAV particles, it was initially questioned if they differ from classically studied enveloped viruses, since the surrounding lipid bilayer of quasi-enveloped particles appears to be devoid of any viral proteins (Feng et al., 2014). Additionally, both types of HAV virions are equally infectious in spite of the apparent absence of any HAV non-structural proteins in quasi-enveloped HAV virions (Feng et al., 2013). Therefore, the question must be asked regarding how membrane-wrapped HAV particles can infect cells in the absence of virus-encoded quasi-envelope-embedded peplomers (Feng et al., 2014). Nevertheless, for HEV, the story appears to be different. It was observed very early (before the observation of quasi-enveloped particles) that monoclonal antibody (mAb) against the HEV-ORF3 product is able to capture HEV particles from sera or cell culture supernatants of infected cells. It is now known that HEV-ORF3 of virus *in vivo* or in cell culture is associated with the lipid component of quasi-enveloped virions, while virions in feces fail to be captured by this mAb because they lack the envelope layer containing HEV-ORF3 (Takahashi et al., 2008). This explanation was recently confirmed by demonstration that immunogold-labeled mAb against pORF3 binds quasi-enveloped HEV particles, as viewed by electron microscopy (Nagashima et al., 2017). Moreover, although infectivity is equivalent between both quasi-enveloped HAV particles and non-enveloped HAVs, quasi-enveloped HEV particles infect fresh cells much less efficiently and require a longer inoculation time to reach maximal infectivity than do non-enveloped HEV particles (Yin et al., 2016). In addition, quasi-enveloped HEV enters cells by endosomal trafficking, which can be abrogated by blocking endosomal acidification (Yin et al., 2016). This additional cell entry mechanism may explain the differences in distinct neutralization mechanisms observed against the two types of HEV particles.

Previously, HEV-ORF3 did not receive much attention, since it overlaps with ORF2 but is translated using an alternate reading frame that encodes a unique protein with little similarity to any other known proteins. Originally, most HEV-ORF3 researchers viewed the ORF3 product as an accessory protein that regulates host signaling to facilitate virus replication and invasion. This hypothesis was supported by evidence demonstrating that HEV-ORF3 is not required for HEV-RNA replication *in vitro* (Emerson et al., 2006b). Subsequently, numerous research studies have also demonstrated that HEV-ORF3 influences regulation of multiple cell signaling pathways involved in host innate immunity (Krain et al., 2014; Nan et al., 2014; He et al., 2016; Nan and Zhang, 2016). Consequently, such regulatory effects may contribute to both viral replication and pathogenesis. In addition, HEV-ORF3 has been shown to be essential for HEV infection *in vivo*, which suggests that HEV-ORF3 must participate in key pathways that affect viral replication. Although several studies suggest that the HEV-ORF3 product is involved in viral release and formation of quasi-enveloped HEV virions (Nagashima et al., 2011a,b), a clear definition of HEV-ORF3’s function has remained elusive until recently.

Very recent HEV-ORF3 studies have shown that the protein shares several structural features with class I viroporins and functions as an ion channel involved in release of infectious particles from cells (Ding et al., 2017). Indeed, the role of HEV-ORF3 during viral replication may be similar to that of a well-characterized viroporin, influenza A virus matrix-2 protein (Ding et al., 2017). As additional clues to ORF3 function, the N-terminal half of HEV-ORF3 contains two hydrophobic domains, of which the first is essential for microtubule association (Kannan et al., 2009) while a putative transmembrane region overlapping with a second hydrophobic domain appears to be involved in ER localization of ORF3 protein (Ding et al., 2017). This study also confirmed that two PXXP motifs (aa 86–89 and aa 95–98) are essential for HEV egress and pORF3 ion channel function (Ding et al., 2017). The latter observation is interesting because viroporins are known to be components of other virions (e.g., matrix-2 protein of influenza A virus) and may help to explain why quasi-enveloped HEV virions contain ORF3 proteins.

Indeed, the presence of HEV-ORF3 protein within quasi-enveloped HEV virions has been confirmed by several studies using anti-pORF3 antibodies to capture quasi-enveloped HEV virions (Takahashi et al., 2008). Therefore, it would be interesting to determine whether anti-pORF3 antibody neutralizes quasi-enveloped HEV virions, especially since anti-ORF2 antibody fails to do so. Although a novel vaccine candidate has been developed based on genotype 4 HEV-ORF3 fused to interleukin-1β and expressed in *E. coli* (Ma et al., 2009), this vaccine only confers partial protection (Ma et al., 2009). This result is consistent with our observations regarding the ORF3 product of avian HEV as a vaccine candidate for chickens (Syed et al., 2017). Subsequently, chickens immunized with bacterially expressed avian HEV-ORF3 protein exhibited milder disease symptoms than did controls upon challenge, thus showing partial protection as well (Syed et al., 2017). However, these vaccine challenge experiments used HEV challenge stocks that were all isolated from fecal samples containing naked HEV virions and free of ORF3 product-associated envelope (Syed et al., 2017). Therefore, during challenge experiments, pORF3-induced antibodies were not able to prevent first-round infection, as they could not neutralize non-enveloped HEV. However, the partial protection rate observed in both experiments may be explained by the speculation that pORF3-induced antibodies are able to neutralize newly packaged quasi-enveloped HEV particles released into circulation after initial infection by non-enveloped challenge virus, thereby reducing disease progression. These observations and speculations lead to a more interesting question: if quasi-enveloped HEV particles are used for challenge, could a pORF3-based vaccine elicit better protection than the current pORF2-based vaccine? This question should be addressed in future investigations to provide valuable information for development of an improved HEV vaccine.

**FIGURE 3:**
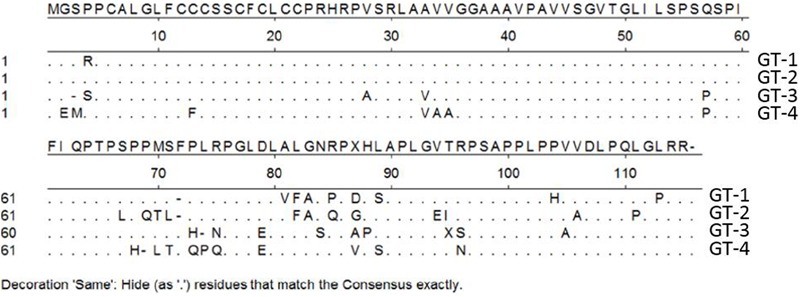
Alignment of amino acid sequence of pORF3 of four HEV genotypes. Genotype 1 (GT-1) (GenBank accession number AF444002), GT-2 (accession number M74506), GT-3 (accession number HQ709170), and GT-4 (accession number AB074915). “**⋅**” represent those residues that are the same as consensus sequence.

Regarding its potential as a vaccine candidate, it appears that the HEV-ORF3 product (including the ORF3 protein of avian HEV) is also highly immunogenic, with most of its epitopes located at the C-terminus (Zhao et al., 2013). Soon after the discovery of HEV and its putative ORFs, research focusing on pORF3 antigenicity and epitope mapping demonstrated that the last 32 aa of HEV-ORF3 comprise an immunogenic region. Subsequently, an artificially synthesized peptide corresponding to this region was reactive with anti-HEV sera from recovered hepatitis E patients (Semiletov et al., 1995; Dement’eva et al., 1997). These results mirror those of our previous report that focused on the use of HEV-ORF3 to enhance type I IFN induction. In that study, rabbit anti-ORF3 antibodies were raised against a peptide located within the C-terminus of genotype 1 Sar-55-ORF3 with a very high binding affinity to the target (detectable at a 1:10000-fold dilution via western blot) (Nan et al., 2014). Therefore, the C-terminal half of pORF3 (about 60 aa) appears to be immunogenic and is a good candidate for a subunit vaccine. However, one major obstacle to development of a pORF3-based vaccine is the antigenic variation of pORF3 across HEV genotypes. Based on the alignment of pORF3 aa sequences of the four major genotypes (**Figure 3**), the C-terminus of pORF3 is not highly conserved (Nan et al., 2014). Meanwhile, our previous research also demonstrates that a genotype 1-specific linear epitope is located within a proline rich region of pORF3 (Sar-55 strain) (Yang et al., 2016), which suggests that there are genotypic differences in pORF3 antigenicity. Thus, it appears that a more systematic mapping of antigenicity epitopes of pORF3 across HEVs may be required for the development of HEV subunit vaccines containing pORF3-derived peptides.

## Conclusion and Future Perspectives

More than two decades have passed since the discovery of HEV and the characterization of its genome. In spite of numerous efforts during that time, our understanding of HEV still remains limited. Nevertheless, ongoing research continues to uncover new information regarding its biology and pathogenesis. However, as a further complication, HEV is no longer viewed as a public health concern only in developing countries, but is now an increasing concern in developed countries due to its complex mechanisms of transmission.

Hecolin^®^, a prophylactic vaccine based solely on the capsid protein of a genotype 1 HEV isolate has been approved in China. However, it is still uncertain whether this vaccine will offer protection against zoonotic HEVs in humans or domestic animals due to new knowledge of cross-species transmission and zoonotic HEV host tropism. Moreover, recently discovered quasi-enveloped HEV particles may partially explain why vaccines targeting capsids have not worked, while also reshaping our understanding of HEV biology and vaccine design. Fortunately, ongoing research is revealing more and more valuable information regarding pORF3 function and is generating optimism that pORF3 could serve as a new target of vaccine design against quasi-enveloped HEV particles.

Across HEV genotypes, ORF3 sequence homology is lower than for ORF2, especially within the immunogenic domain of approximately 66 aa within the ORF3 C-terminus. It is hoped that epitope scanning and analysis may soon help to identify major neutralizing epitopes against quasi-enveloped HEV virions. Subsequently, if neutralizing epitopes from different HEV genotypes could be identified, full-length pORF3 may not be needed as an immunogen. Instead, a combination of peptides containing neutralizing epitopes from various HEV genotypes could be explored as a novel peptide vaccine. Such a vaccine might serve as a booster shot for the current pORF2-based subunit vaccine to ultimately provide better protection than from the pORF2 vaccine alone. Using these new strategies, it is hopeful that the next generation of HEV vaccines will finally achieve solid cross-protection against zoonotic HEV.

## Author Contributions

All authors listed have made a substantial, direct, and intellectual contribution to the work, and approved it for publication.

## Disclaimer

Mentioning of trade names or commercial products in this article is solely for the purpose of providing specific information and does not imply recommendation or endorsement.

## Conflict of Interest Statement

The authors declare that the research was conducted in the absence of any commercial or financial relationships that could be construed as a potential conflict of interest.
